# From Pomegranate Byproducts Waste to Worth: A Review of Extraction Techniques and Potential Applications for Their Revalorization

**DOI:** 10.3390/foods11172596

**Published:** 2022-08-26

**Authors:** Marina Cano-Lamadrid, Lorena Martínez-Zamora, Noelia Castillejo, Francisco Artés-Hernández

**Affiliations:** 1 Postharvest and Refrigeration Group, Department of Agronomical Engineering and Institute of Plant Biotechnology, Universidad Politécnica de Cartagena, 30203 Cartagena, Spain; 2 Department of Food Technology, Nutrition, and Food Science, Faculty of Veterinary Sciences, University of Murcia, Espinardo, 30071 Murcia, Spain

**Keywords:** *Punica granatum*, circular economy, sustainability, antioxidants, phenolics, encapsulation, green-technology, minimally processed, food losses, clean label

## Abstract

The food industry is quite interested in the use of (techno)-functional bioactive compounds from byproducts to develop ‘clean label’ foods in a circular economy. The aim of this review is to evaluate the state of the knowledge and scientific evidence on the use of green extraction technologies (ultrasound-, microwave-, and enzymatic-assisted) of bioactive compounds from pomegranate peel byproducts, and their potential application via the supplementation/fortification of vegetal matrixes to improve their quality, functional properties, and safety. Most studies are mainly focused on ultrasound extraction, which has been widely developed compared to microwave or enzymatic extractions, which should be studied in depth, including their combinations. After extraction, pomegranate peel byproducts (in the form of powders, liquid extracts, and/or encapsulated, among others) have been incorporated into several food matrixes, as a good tool to preserve ‘clean label’ foods without altering their composition and improving their functional properties. Future studies must clearly evaluate the energy efficiency/consumption, the cost, and the environmental impact leading to the sustainable extraction of the key bio-compounds. Moreover, predictive models are needed to optimize the phytochemical extraction and to help in decision-making along the supply chain.

## 1. Background—Food Losses and Food Waste

In accordance with the Food and Agriculture Organization of the United Nations (FAO) definition, ‘food waste’ is the decrease in the quantity and/or quality of food obtaining from decisions and/or actions of retailers, food service providers, and consumers, while ‘food loss’ refers to any food that is discarded along the food supply chain, from harvest up to retail sale [1]. FAO indicates that around one third of global food production is lost or wasted at some step in the food chain. The degree of loss greatly varies depending on the state and the basket item.

**Figure 1:**
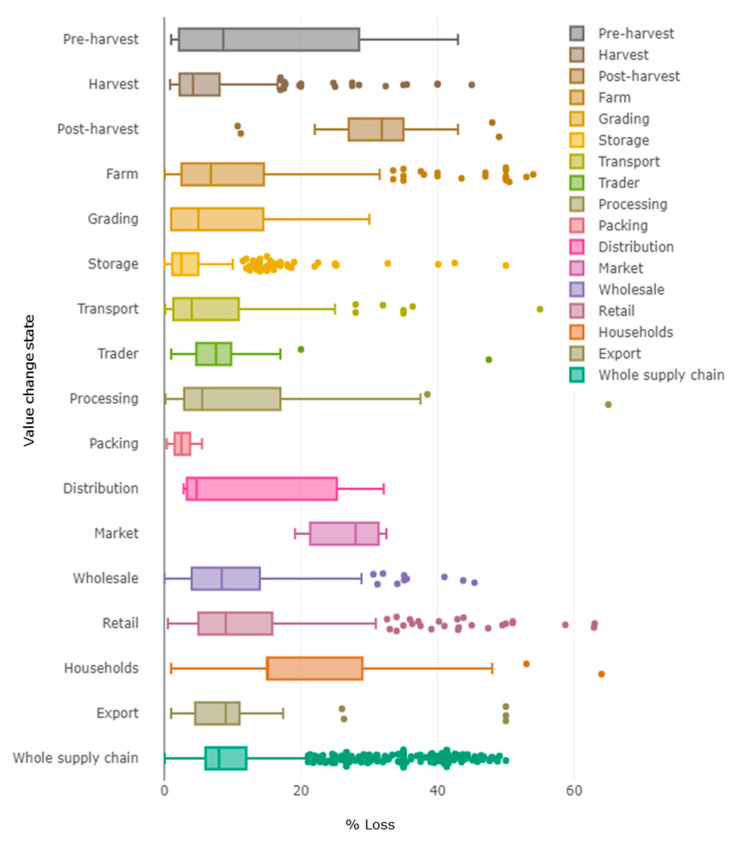
Food loss (%) of F&V in the world by Food Loss and Waste Database (FAO). Year range: 2000–2020; Aggregation: World; Basket items: Fruit and Vegetable; Country: All; Method data collection: all. Reprinted/adapted with permission from Ref. [2]. Copyright year: 2022; copyright owner’s name: FAO.

In the case of fruit and vegetables (F&V), losses over the whole supply chain could reach up to ~50% (Figure 1). FAO’s future challenge is to reduce ~50% of food waste by 2050, as one of the objectives for sustainable development (OSD). The circular economy has been considered as the principle for eco-innovation, being focused on a ‘zero waste’ society and economy, using wastes as raw materials.

Between 2016 and 2018, FAO Statistics Division developed a food loss estimation model called ‘*The Food Loss and Waste database*’, an online collection of data including food loss and food waste. Figure 1 shows the percentage loss of F&V (food loss + food waste) worldwide in each value chain step for the first 20 years of the twenty-first century [2]. The boxes show where ~50% of the collected data falls into, and the mid-value of the percentage loss at every stage in the supply chain is shown by a line. In this sense, postharvest and retailing are the steps in the food chain where the F&V losses represent the highest mean percentages. The mean percentage during processing is less than 10%, but in some cases, it reaches ~40%. Moreover, although the mean percentage during distribution represents less than 10%, the range is from <5% to >30%. Therefore, several strategies have been developed around the creation of active packaging with encapsulated key compounds, to avoid the high percentage of food waste/loss [3]. The range of loss percentages at each step is wide since the value depends on the type of F&V, the country, and the year.

Although this review is focused on pomegranate byproducts, the percentage of food loss related to this fruit is not available in the mentioned official database. Nevertheless, knowing that the total production of pomegranate worldwide is three million tons, and its peel and seeds represent ~54% of the fruit, this results in ~1.62 million tons of waste [4,5]. Therefore, it is a huge amount of waste produced, so it is crucial to find suitable methods to revalorize it by optimizing the bioactive compounds extraction of pomegranate residues, and then converting them into value-added products. Consequently, savings can also be made on other resources involved during production, harvesting, preservation and distribution, such as energy, water, and land, as well as contributing to the environment [5]

Health, well-being, and sustainability are the current trends in the food market. Consumers and food producers are interested in ‘clean label’ foods or ingredients [6,7]. It means that they are interested in foods or ingredients obtained by green processing technologies (non-thermal, green solvent), and bioactive compounds with health promoting properties (nutraceuticals), among others. The bioactive compounds obtained from F&V byproducts present technological and functional features that can be incorporated within other food matrixes to enhance their nutritional, functional, and sensory quality [6,8]. Moreover, bioactive compounds from F&V byproducts have previously been classified as potential green ingredients for the cosmetic and pharmaceutical industries, and used in developing different products intended for specific populations, such as sportspeople [9].

The present review aims to evaluate the scientific evidence and knowledge on the use of green technologies for the extraction of phenolic compounds from pomegranate byproducts, and the incorporation techniques and potential applications via the supplementation/fortification of F&V matrixes to improve their quality and safety in a circular economy. For this purpose, a literature review was conducted, focusing on ultrasound-, microwave-, and enzymatic-assisted technology to enhance phenolic compounds extraction from pomegranate peel byproducts. Moreover, different incorporation techniques and applications have been reviewed. The results may provide the scientific community with an overview of the state of the art in pomegranate peel revalorization. The study may also help scientists and the food industry to develop solutions to better suit society’s demands.

## 2. Nutritional Composition of Pomegranate Byproducts

**Figure 2:**
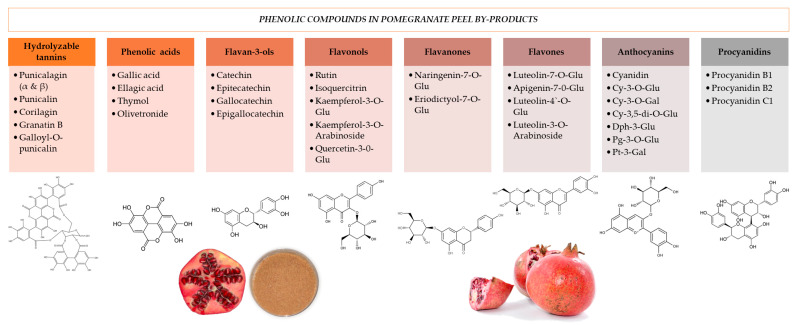
Classification of the main phenolic compounds in pomegranate peel [5,15]. Glu: glucoside; Cy: cyanidin; Dph: delphinidin; Pg: pelargonidin; Pt: petunidin; Gal: galactoside.

Both primary (sugars, pectins, proteins, and fats) and secondary (polyphenols, pigments, and sulfur compounds) metabolites have been found in F&V byproducts [10]. The food industry and researchers are interested in reducing the environmental impact, and then focus on the recovery of the target compounds [6]. Carbohydrates (around 60%) [11], pectin (yield range from 6 to 25%) [12,13], proteins (around 3%) [14,15], and fats (<1%) [15] have been previously identified in pomegranate peel. Since this review is focused on the extraction of secondary metabolites from pomegranate peel, especially phenolic compounds, Figure 2 shows the classification of the main ones found [5,15].

Among them, the top ten have recently been identified and quantified [16], being punicalagin (28,000–104,000 µg/g) the major compound found, followed by ellagic acid (1580–4514 µg/g), and others such as punicalin (203–840 µg/g), catechin (115–613 µg/g), corilagin (71–418 µg/g), gallic acid (10–73 µg/g), gallocatechin (69–1429 µg/g), epigallocatechin (5–106 µg/g), epigallocatechin gallate (4–70 µg/g), and kaempferol-3-O-glucoside (16–99 µg/g) [16].

Apart from pomegranate peel, seeds (wooden part) are generated after juice processing as a byproduct. Although this review is not focused on pomegranate seeds revalorization, previous studies have indicated that pomegranate seeds are rich in polyunsaturated fatty acids (88–92%), the most abundant being linolenic acid, especially punicic acid which ranges in terms of percentage of total fatty acid profile from 59.7 to 74.3% [17,18]. 

## 3. Scientific Literature Review

This review has been written as a research paper. Thirty-seven studies related to ultrasound-, microwave- and enzymatic-assisted extraction of phenolic compounds from pomegranate peel were collected using the PRISMA Extension (PRISMA-ScR) approach for scoping reviews. In a similar way, 21 and 28 studies were included on incorporation techniques and potential applications, respectively.

A comprehensive literature search using Web of Science and Scopus was performed in June–July 2022. Text words (pomegranate, peel, byproduct, application, ultrasound-, microwave- and enzymatic-assisted extraction) within the titles, abstracts, and keywords, were used. Original research papers and reviews with experimental design and data treatment in journals included in Journal Citation Reports (JCR) were selected.

## 4. Pomegranate Peel Phenolic Compounds Extraction Techniques

Conventional technologies are still in use, although these entail high energy consumption, and thermolabile nutritional compounds degradation during the process. Green extraction technologies have recently been developed using current technologies. These technologies use fewer non-green solvents, minimizing environmental and health impacts. Moreover, selective extraction is important for the bioactive compounds yield. Industry and research are focused on green extraction methods such as ultrasound-, microwave-, pulsed electric field- and enzyme-assisted extractions, among others [19].

Additionally, it is worth mentioning that processing, including drying (i.e., convective or freeze drying), homogenization, and/or grinding into powder are used as pre-treatments of extraction techniques. Even enzymatic treatment is classified as a pre-treatment of extraction processing. The drying method used for byproducts as a pre-treatment for extraction also needs to be optimized, as many of the bioactive compounds are degraded during drying. The technique, the time, and the temperature should be selected to avoid the degradation of the compounds and to have a stable material (dry byproduct) for storage until the extraction. Therefore, this process is of great importance for obtaining the best quality extracts. Depending on the drying process, the moisture content of the sample varies and influences the extraction step. Previous studies have indicated that particle size is one of the critical parameters affecting the extraction. The reduction by grinding could increase the diffusivity of the bioactive compounds, and promotes the rupture of the cell walls. Moreover, several authors indicate that blanching F&V byproducts as a pre-treatment could be a good strategy to enhance the recovery of phenolics during pomegranate peel extraction [4].

In this review, we are focused on ultrasound-assisted extraction (UAE), microwave-assisted extraction (MAE), and enzyme-assisted extraction (EAE) technologies. In the following section, the most important parameters of each technique are defined.

### 4.1. Ultrasound-Assisted Extraction

#### 4.1.1. Fundamentals

Ultrasound (US) means mechanical waves propagated in an elastic medium through the transfer of energy and not of particles, to induce the longitudinal displacement of particles [20,21]. This consists in a succession of phases: (i) compression, and (ii) rarefaction, into the medium. If the strength of the rarefaction cycle is sufficient, the critical molecular distance of the liquid can be reached, and cavitation bubbles are created, creating the effect of US. The cavities increase and decrease in size during the contraction and compression phases, respectively. The bubbles generated could reach a great size, collapsing and generating large amounts of energy. The temperature and pressure at the collapse moment have been calculated to be up to 4727 °C and 5000 atm in an ultrasonic bath (25 °C) [22]. These bubbles collapse onto the surface of a solid material, and the high pressure and temperature reached create microjets directed towards the solid surface. These microjets are used in the food industry for the key bioactive compounds extraction, destroying the cell walls of the plant matrix, and its content can be released into the medium. The main parameters influencing the US technique are described below.

*Type*

There are two main US types: bath and probe. The first one consists of a stainless-steel tank with one or more ultrasonic transducers. The US intensity distribution is heterogeneous; therefore, the container must be located at the position where the highest intensity of sonication is achieved. The US probe is a great tool for the solid–liquid extraction of bioactive compounds. The shape and the diameter of the probe are the main characteristics that have an impact on bioactive extraction. Both US types can be applied in different modes: continuous, sweep, and pulsed mode. The main differences between the types are:

i Contact with the solution: an ultrasonic probe is submerged directly into the solution (minimum energy losses), while in a US bath, the vessel container is immersed.
ii Intensity of US: it is higher in the US probe than in the bath.
iii Maximum power achieved: in a US probe, it is the nominal power, while in a US bath, the nominal power is the minimum that can be increased due to the modulators.

*Frequency*

US frequency is expressed in Hertz (1 Hz ≈ 1 cycle/s). In a US process, the use of ultrasonic waves in the range from 20 to 100 kHz is common, and the concept time of one cycle means s/Hz.

*Power/Energy Intensity/Density (Dose)*

US power is expressed in watts (W), being a key parameter to express the efficiency of the process. The amount of energy applied in the system could be expressed as ultrasonic intensity (energy per second and per square meter of emitting surface, expressed in W/s o W/cm^2^) or acoustic energy density (the amount of US energy per unit volume of sample, expressed in W/cm^3^ or W/mL).

*Amplitude level (A)*

The amplitude of a wave is the height of the wave and is expressed in µm. It is important to clarify that in a US probe, the term amplitude level is commonly used. The amplitude control of the processor allows to set the ultrasonic vibrations at any desired level in the 10–100% range of the nominal power.

*Pulse duration/interval ratio (Duty cycle)*

This parameter is used in the pulsed US process. Pulse duration is the time for which the ultrasonic probe is *on*; pulse interval indicates the time for which ultrasonic probe is *off*; and cycle time is the sum of pulse duration and pulse interval. Duty cycle is the main way to express this parameter and can be expressed as a ratio (pulse duration/cycle time) or percentage ((pulse duration/cycle time) × 100).

*Temperature*

This parameter is important for the efficiency of bioactive compounds extraction. Although previous studies have indicated that an increase of temperature means an increase of extraction yield, it is essential to select an extraction temperature. The main reason is that some possible key bioactive compounds are thermolabile. Therefore, temperature optimization is needed to obtain the highest extraction yield of the key bioactive compound.

*Extraction time*

As with the temperature and power parameters, increasing the time in the early stages of the US process increases the extraction yield, whereas a decrease in the yield is usually observed as the extraction time increases. At the beginning, the cavitation effect of the US increases the swelling and hydration. Both swelling and hydration could be achieved by shaking. Later, the fragmentation and pore formation of the plant tissue matrix occurs, extracting the key bioactive compounds. Excessive US exposure causes structural damage to the solute and decreases the extraction yield, and even the degradation of the extracted bioactive compounds.

*Solvent*

The selection of the solvent for US extraction depends on the target bioactive compound. It is essential to consider the physicochemical properties of the solvent and the bioactive compounds such as viscosity, pH, surface tension, and vapor pressure of the solvent, as well as the solubility of the key bioactive compounds. The most common solvents used during US extraction are water, ethanol, alcohols, and acetone in different concentrations. Also, the concentration and the solid–liquid ratio are important factors affecting the extraction yield and properties of the bioactive compound during UAE.

#### 4.1.2. Ultrasound-Assisted Extraction from Pomegranate Peel

Apart from the variables described above, there are other variables specific to the raw material that should be considered, such as cultivar, drying, moisture content, and particle size. Although pomegranate peel is a large reservoir of bioactive compounds, the variability of the amount and profile depends on the cultivar selected [23]. However, no cultivar information is available in 45% of the published studies, and just one of them compared two cultivars (both sour cultivars: Wonderful and Akko) with the same methodology of drying and extraction (Table 1).

Table 1 shows the different conditions of drying in all studies related to US, except one in which no information is available and one in which fresh pomegranate peel was used. Taking all the studies into account, the temperature range used is from 25 °C (room temperature) to 70 °C. On the other hand, particle size was not indicated in more than 10% of the studies showed in Table 1, and the range is from 800 µm to 25.4 mm. Moreover, one study indicated that a paste of pomegranate peel was used, and two studies described a combination of particle size in which the distribution was indicated.

The frequency range is between 20 and 80 kHz, with 20 kHz being the most common frequency used (30% of the studies included in Table 1). The power parameters were not unanimous due to the different information described: units (power, power density), the equipment used (bath, probe), and the mode (continuous, pulsed). The range of power was from 50 to 1050 W, while power density was 0–1600 expressed in W/L, and from 2.4 to 59.2 in W/cm^2^. More details related to the US probe or US bath are included in “other information”, such as probe diameter, submerged distance, and duty cycle. Although ethanolic solvents, with different percentages of ethanol, were mostly used (>50% of the studies included in Table 1), other authors selected solvents such as acetone, water, and methanol. It is essential to clarify that polar solvents (mainly water) extract more hydrophilic compounds (which only participate in reaction with oxygen–hydrogen bonds as sugars), and ethanolic solvents (which participate in reaction with oxygen–hydrogen and polar carbon–oxygen as ethanol) are more effective in extracting phenolic compounds [24]. The solvent changes depending on the target bioactive compounds; for instance vegetal oil was used as a solvent to extract carotenoids. The solid–liquid ratio was included in all the studies, and was highly variable among them. Information on the time and the temperature used during US extraction was lacking in about 20 and 30% of the studies, respectively. The range of time and temperature included was 0–240 min and 20–93 °C, respectively.

The target individual compounds found were punicalagin (Pn), individual phenolics (Ph), individual tannins (Tn), ellagic acid (EA), chlorogenic acid (ChlA), gallic acid (GA), individual flavonoids (Fvs), and hydroxybenzoic acids (HbA). The importance of other bioactive compounds from pomegranate as targets to optimize the extraction process, such as anthocyanins and alkaloids, should be noted as being of interest for the food, pharmacological, and cosmetic industries. In addition, spectrophotometric techniques were used to determine the yield of the extraction, the total phenolic content (TPC), total flavonoid content (TFC), antioxidant activity (AOX), total anthocyanin content (TAC), and total carotenoids content (TCC). Undesirable compounds formed during the treatment, such as hydroxymethylfurfural as a furan derivative, could be a good strategy to optimize the process.

Regarding the best conditions for extracting bioactive compounds from pomegranate peel, different optimal processing conditions can be found in the published scientific manuscripts. Following the literature review, some of the optimized conditions are presented below. The highest (506 mg g^−1^ dw) punicalagin content was obtained by UAE with 53% EtOH, s solid–liquid ratio of 1:25 *w*/*v*, and power at 757 W for 25 min [25]. More and Arya (2021) [26] concluded that the optimum processing conditions were 2:100 solid–liquid ratio, 116 W (80% duty cycle) for 6 min, obtaining a 0.48 g/g yield, and a TPC of 178 mg GAE/g dw. Pan et al. [27] observed that pulsed US extraction with 59 W/cm^2^, and 5/5 of pulse duration/interval duration increased the antioxidant yield (22%) and reduced the extraction time (87%) compared with conventional extraction. Furthermore, when the US extraction was continuous with the same conditions, the antioxidant yield increased by 24% and reduced the extraction time by 90% [27]. Other authors have reported that US increased the extraction yield reducing by 20-fold the time required [28]. Moreover, the extraction yield increased with increasing extraction temperature from 25 to 35 °C [28]. Other research has proposed a mathematical model for multi-criteria optimization to enable the prediction of bioactive compounds extraction for any temperature (20–60 °C), solvent (0–100% ethanol), and US power density (0–100 W/L), at any time (0–240 min). This model reveals the optimal conditions for obtaining the best yield of the target compound with the minimum time and/or energy consumption [29]. For the recovery of ellagic acid, Muñiz-Márquez et al. [30] indicated that the best extraction conditions were at 94 °C for 55 min using ethanol 75% and 1:3 solid–liquid ratio [30]. To recover carotenoids, the most efficient extraction period to achieve the maximum yield from pomegranate peel was about 30 min with the following conditions: 52 °C, 0.10 solid–liquid ratio, amplitude of 58.8%, and sunflower oil as a solvent [31].

**Table 1:** Ultrasound conditions (frequency, power parameters, solvent, time, temperature) for the extraction of bioactive compounds from pomegranate peel.

Byproduct Characteristics	F (kHz)	Power Related Parameters	Solvent	Solid:Liquid Ratio	t (min)	T (°C)	Other Information	Extract Characterization	Ref.
Freeze-drying Powders < 254 µm cv information NA	NA	500–1050 W	EtOH (40–80%)	1:10/1:50	10–50	NA	NA	Pn	[25]
Vacuum oven (45 °C, 36 h) Powders < 500 µm Bhagwa cv	NA	350 W (Pulsed mode: A 10–100%)	EtOH (50%)	0.1:10/0.3:10 0.5:10/0.6:10 0.8:10/1:10	NA	NA	Duyt cycle: 10–90%	TPC, TFC, AOX (DPPH and ABTS), TAC	[26]
Cabinet hot drier (40 °C) Powders < 635 µm cv information NA	20	2.4 to 59.2 W/cm^2^ (continuous) 59.2 W/cm^2^ (pulsed)	NA	1:50	2–90	25	Area probe: 1.267 cm^2^ Pulse duration/Interval: 2/1, 3/1, 4/1, 5/1, 6/1, 7/1, 9/1, 12/1, 2/5, 3/5,4/5, 5/5, 6/5, 7/5, 9/5, 12/5, 2/15, 3/15, 4/15, 5/15, 6/15, 7/15, 9/15, 12/15 Number of pulse repetition: 30, 60, 90, 120, 180, 270, 360, 540, 720	TPC, AOX (DPPH)	[27]
Drier (40 °C, 48 h) cv and particle size information NA	20	130 W (Pulsed mode: A 20 and 60% NP)	MetOH, EtOH, EtAc, MeOH (50%)	1:10/1:50	4–60	25–45	Ti–Al–V sonoprobe (13 mm) Pulse duration/pulse interval ratio 5/15 and 2/1	Ph	[28]
Vacuum oven (40 °C) Powder 125–150 μm cv information NA	30	50 W (A 30–70% NP)	EtOH (80%)	1:40/1:120	5–50	NA	Duty cycle (60–100%) Probe diameter 3 mm Length 80 mm	Pn	[32]
Air oven (40 °C, 18 h) Powders 3 mm cv information NA	30.8	0–100 W/L	EtOH (0–100%)	1:40	0–240	20–60	NA	TPC, AOX (DPPH)	[29]
Microwave vacuum oven (45 °C, 36 h) Powders < 508 µm Kabuli cv	20	700 W (A 40–80%)	Ac (30–90%)	1:10/1:30	10–20	45	Probe half-inch diameter Pulse-on and pulse-off time of 10 s.	Tn	[33]
Air oven (60 °C, 48 h) Powder 600–800 μm cv information NA	40	NA	EtOH (24–75%)	1:3/1:16	4–55	26–93	NA	EA	[30]
Fresh peel (more information NA) Small-grinded pieces (fine peel paste, 4 °C) cv information NA	20	400–1600 W/L	NA	2:10	5–50	30–70	Pulsed mode: ‘on’ time (5 s) Pulse interval ‘off’ time (3 s) Probe: 3 cm submergence of sonicator	TPC	[34]
Dried (more information NA) Powders < 0.5 mm cv information NA	40	500 W (A 50–80%)	MetOH, (30–70%)	1:15	5–15	40	Probe: 6 mm diameter, dipped up to 2 cm Duty cycle: 0.2–0.8	TPC, TFC, AOX (DPPH and FRAP)	[35]
Laminar airflow drying oven (50 °C, 24 h) Powder particle size and cv information NA	24	NA	NA	1:10	5–20	50	Titanium probe: 14-mm diameter	TPC, EA, ChlA, GA	[36]
Hot air oven (40 °C, 48 h) Fine powder (more information NA) cv information NA	45	360 W (A 40–100%)	NA	0.1:1/1:10	5–45	40–80	pH 3.5 to 6.0	TPC	[37]
Technique information NA Powder 140–425 μm Sishe Kape- Ferdos cv	20	400 W (A 20, 60 and 100%)	NA	1:4	5–15	NA	NA	TPC	[38]
Forced air oven (70 °C, 48 h) Powder particle size distribution: 25.4–0.105 mm (56%); 0.105–0.075 mm (17.9%); 0.075–0.037 mm (14.5%); <0.037 mm (11.6%) Brazilian Molar cv	37–80	180 W (continuous, pulse, and sweep modes)	EtOH (70%)	1:25	20	40–70	NA	Pn, EA, TPC	[39]
Drier (40 °C, 48 h) Powder < 0.2 mm cv information NA	20	130 W (Pulsed mode; 20–60%)	Sunflower oil Soy oil	1:10/3:10	NA	10–60	Ti-Al-V sonoprobe (13 mm)	TCC	[31]
Air-drier (7 days, 20 °C) Powder < 180 μm Malas cv	24	53, 79, and 105 W/cm^2^ (pulse mode)	EtOH (70%)	1:10	2–10	NA	Area probe: 1.53 cm^2^ Duty cycle: 50%, 70%, and 90%	Pn, HbA	[40]
Traditional heating oven (40 °C, 48 h). Microwave drying (T < 100 °C, <5 min). Powder < 150 μm. Wonderful and Akko cv	26	200 W (pulsed mode; A 50%)	EtOH (70%)	1:40	10	45	Duty cycle: 80%	EA, Pn	[41]
Hot air oven (50 °C, 48 h) Fine powder (more information NA) cv information NA	NA	NA	Ac, MetOH, EtOH (50–75%)	1:20	NA	45	NA	TPC, TFC	[42]
Air dried Room Temperature Particle size 0.3 mm cv information NA	20	400 W A 70%	EtOH (70%)	1:30	30	40	Probe tip 2 cm 22.5% duty cycle	TPC, TFC, AOX (DPPH and FRAP)	[42]
Ventilated oven (42 °C, 3–4 days) Particle size 0.5 mm cv information NA	NA	NA	H_2_O; EtOH (70%); EtOH (100%); Ac (70%); Ac (100%);	NA	23	45	NA	TPC, EA, Pn, Individual Fvs	[43]
Convective oven 60 °C 22 h Particle size 420 μm Mollar de Elche cv	20	750 W	H_2_O	4:40	NA	NA	Probe diameter 13 mm titanium	TPC, AOX (DPPH and ABTS)	[44]
Blanching (80 °C 3 m) + Tray drier 40 °C Particle size < 1 mm Wonderful cv	40	700 W	EtOH (70%)	1:15	60	40 °C	Ultrasound bath	TPC, TFC, TAC, Vit C, AOX (DPPH, FRAP and ABTS)	[45]
Tray drier 40 °C Particle size < 0.25 mm Bhagwa cv	20	20–40% A	EtOH (70%)	1:20	10–20	40–60	3 mm of probe diameter	Pn, EA, GA	[46]
Oven drier (more information NA) Particle size: size distribution using sieves: 0.85, 0.425, 0.25 and 0.18 mm cv information NA	35	140 W	EtOH (30–50–70%)	0.2:10	10–30	30–60	Ultrasound bath	TPC, AOX (FRAP and DPPH)	[47]
Oven drier (45 °C 48 h) Particle size and cv information NA	40	NA	NA	1:10	0–60	35–45	Enzymatic pre-treatment	AOX (DPPH), TPC	[48]
Hot air in cabinet drier (40 °C) Particle size < 0.420 mm Wonderful cv	20	Continuous intensity: 2.4, 4.7, 7.1, 18.9, 23.7, 30.8, 37.9, 45.0, 52.1, and 59.2 W/cm^2^	H_2_O	1:50	2–90 min	25	Probe with area of 1.267 cm (continuous)	AOX (DPPH)	[27]
Hot air in cabinet drier (40 °C) Particle size < 0.420 mm Wonderful cv	20	Pulsed mode: 2.4, 4.7, 7.1, 18.9, 23.7, 30.8, 37.9, 45.0, 52.1, and 59.2 W/cm^2^	H_2_O	1:50		25	Probe with area of 1.267 cm Pulsed duration/interval: 2/1, 3/1, 4/1, 5/1, 6/1, 7/1, 9/1, 12/1, 2/5, 3/5, 4/5, 5/5, 6/5, 7/5, 9/5, 12/5, 2/15, 3/15, 4/15, 5/15, 6/15, 7/15, 9/15, 12/15 Number pulse repetition: 30, 60, 90, 120, 180, 270, 360, 540, 720	AOX (DPPH)	[27]
Air-dried at 25 °C Particle size 0.75–2 mm cv information NA	NA	NA	EtOH (10–30–50–70–90%)	1:10; 1:20; 1:30; 1:40; 1:50	5–65	25–80		Pn, EA, GA, TPC	[49]
Forced air oven 70 °C 48 h Large particle size: 0.297–1.410 mm, mean: 1.05 mm Small particle size: 0.177–1 mm, mean: 0.68 mm Wonderful cv	19	0–800 W at the generator, or 0–38.5 W at the tip of the probe	H_2_O; EtOH (30–50–70%)	NA	10	50–100	13 mm diameter probe Number of Cycles: 5 Assisted by pressurized liquid	Pn, EA, P	[50]

NA: Data not available; cv: cultivar; A: amplitude; F: frequency; NP: nominal power; Ac: acetone; Pn: punicalagina; P: punicalin; TPC: total polyphenolic content; TFC: total flavonoid content; AOX: total antioxidant capacity; TAC: total anthocyanin content; Ph: phenolics; Tn: Tannins; EA: ellagic acid; ChlA: chlorogenic acid; Fvs: flavonoids, GA: gallic acid; TCC: carotenoids; hydroxybenzoic acids (HbA); Fvs: flavonoids.

US treatments are also combined with other green technologies to increase the extraction effectiveness. With regard to pomegranate peel, US treatment was jointly applied with different combinations of expansion gas initial pressure [51] and system pressure [52]. These results suggest the great potential of expansion gas in pressurized liquids assisted by US using green solvents for the extraction of polyphenols [51]. Another technology used in combination with US for bioactive compounds extraction from the pomegranate peel was the extraction at the cloud point, the combination studied being the one that gave the maximum extraction of polyphenol and flavonoid content [52]. More information on the combination with EAE technology is given in Section 4.3.

### 4.2. Microwave-Assisted Extraction

#### 4.2.1. Fundamentals

Microwave (MW) energy is based on electric and magnetic fields, obtaining electromagnetic waves. This energy is non-ionizing, facilitating molecular movement by ion migration and dipole rotation without altering the molecular structure, generating friction and then heat. MAE is based on the disruption or changes in the structure of cells when applying non-ionizing electromagnetic waves to a sample matrix [53]. Performance in MW-assisted processes is highly influenced by several variables. Therefore, the main characteristics and parameters are described below [54,55].

*Pressure*

For MAE, the most common instrument is a closed-vessel system in which the pressure and the temperature can be modulated, and then optimized to accelerate the mass transfer of target compounds from the sample matrix, avoiding degradation [56].

*Power Intensity/Density (Dose)*

In a similar way to the US technique, one of the most important factors to be considered in the MAE is the MW power density, expressed as the power to be applied to the product per unit weight or volume. An increase of the MW power enhances the penetration of solvent into the solid, and then the extraction and recovery of bioactive compounds. Power should be selected to optimize yields and for the selectivity of the desired components, without affecting their stability.

*Temperature*

An increase in temperature during extraction promotes an increase of the diffusivity of the solvent into the solid, and then a desorption of the target compounds occurs. Temperature control is carried out by a probe. Focusing on avoiding undesirable changes, the extraction temperature should be selected considering the stability and extraction yield of the desired active compounds.

*Time*

As the extraction time increases, the yield increases, but high power during long application times is associated with thermal degradation. Therefore, a combination of low/moderate power with longer exposure may be considered. Depending on the matrix and the target bioactive compounds, the optimal condition changes.

*Solvent*

Considering what has been stated in relation to solvents in the section on US, the viscosity of the solvent affects molecular rotation, and therefore the ability of samples to absorb MW.

#### 4.2.2. Microwave-Assisted Extraction from Pomegranate Peel

Table 2 shows the scientific evidence on MAE of bioactive compounds from pomegranate peel. The drying technique, particle size, and cultivar information used during pomegranate peel from 0.5 mm to 2 mm. The power, temperature, and time used during MW treatment was from 100 to 6000 W, from 40 to 72 °C, and, from 0.5 to 40 min, respectively. Ethanol, methanol, and water were the solvents used during MAE. Different solid–solvent ratios were studied to optimize the process, from 1:10 to 1:60. Apart from the bioactive compounds, it is crucial to focus on other compounds such as hydroxymethylfurfural, when high temperatures are reached during food processing.

In a previous review related to MW extraction of pomegranate peel, several manuscripts were included that are omitted here [4] because they are not in the JCR list. Related to the optimal conditions, a previous study indicated that the optimum parameters of vacuum MAE were 10–12 min, 61–79 °C, 3797–3577 W, and 38–39% ratio of water to raw material (39.92% and 38.2%) to obtain the highest values of TPC (5.5 mg Gallic Acid Equivalent/g fresh pomegranate peel) [57]. Other authors have reported that the optimum operating conditions were extraction with ethanol 50%, 1:60 solid–liquid ratio, and 600 W [58]. The results were compared with those obtained by UAE studied in a previous work by the same research team, concluding that the MW method presented a 1.7-fold higher yield after 4 min than after 10 min by UAE [58]. Regarding phenolic extraction, another study indicated that MAE (low MW power and 50% ethanol) was useful for phytochemical extraction [59]. Although there are relevant and promising results, they are nonunanimous and scarce. Therefore, more research on MAE and comparison with other green techniques is required.

### 4.3. Enzymatic-Assisted Extraction

#### 4.3.1. Fundamentals

Enzyme assisted extraction (EAE) is also classified as a green technique. The purpose of this technique is the addition of enzymes in the extraction medium, usually as a pre-treatment of other techniques to enhance the yield, breaking, and/or softening the cell walls. Therefore, thanks to the digestion of the cell walls, bioactive compounds (bound or dispersed inside the cells and on cell walls) can be directed out of the cell to the solvent [19]. EAE extraction depends on several variables that are described below. Among enzymes, pectinases, proteases, and cellulases (and their combinations) are the most used for the extraction of bioactive compounds from F&V byproducts. Pectinases degrade the pectin present in cell walls, and are mainly used in food industries for the clarification and extraction of fruit juices, emerging as a new tool for the extraction of bioactive compounds [60,61]. Proteases are hydrolase enzymes that digest proteins and peptides, and even hydrolyze peptide bonds present in cell walls [62]. Cellulases are key enzymes in the food industry, as they play an important role in the overall carbon cycle. This is due to the degrading of insoluble cellulose into soluble sugars. It is important to highlight that cellulases are the most diverse type of enzymes, catalyzing the single substrate hydrolysis [63]. According to van Oort [64], the main limiting factors in the reaction speed and enzymatic activity are:

i. Solute concentration
ii. Enzyme concentration: when the enzyme concentration increases, the reaction rate will increase until a point when there is no positive or negative effect of the continued increase in enzyme concentration.
iii. Temperature: the optimum temperature for maximum enzyme activity depends on the type of enzyme since most of them are proteins which are denaturized and inactivated by heat.
iv. pH: enzymes have a characteristic pH value for their optimum activity, being acidophilic (optimal pH values are <7) and alkalophilic enzymes (pH > 7). At pH values greater or lower than the optimal, the enzymatic activity—and therefore the reaction rate—decreases. Furthermore, the optimal value of enzyme concentration, temperature, and pH depends on the conditions of the medium/matrix in which it is found.
v. The presence of inhibitors: molecules that temporarily or permanently interact with enzymes to reduce their activity and/or reaction rate. The inhibition can be classified as:
  a. *Competitive*: the inhibitor structure is like the substrate. The key bioactive compound and the complex substrate-enzyme is not formed.
  b. *Acompetitive*: the inhibitor structure is attached to the complex-enzyme.
  c. *Non-competitive*: the inhibitor attached to the active center of the enzyme and enzyme is misshapen.
  d. *Mixed*.

Apart from the limiting factors of the speed and enzymatic activity, the inactivation protocol is also required to optimize the extraction time. Therefore, all mentioned key parameters should be detailed and optimized. At the end of the EAE, enzyme inactivation is necessary. The inactivation conditions (temperature, time) depend on the enzymes used.

#### 4.3.2. Enzyme-Assisted Extraction from Pomegranate Peel

The detailed information related to the EAE of bioactive compounds from pomegranate peel is shown in Table 3. The enzymes used in the literature were pectinase, protease, and cellulase, while the temperature ranged from room temperature to 45 °C. After enzymatic pre-treatment, hydrolyzed pomegranate peel continues the extraction with other green technologies (high pressure, supercritical carbon dioxide, and ultrasound). Comparing with a previous review [4] which encompassed the enzymatic extraction of pomegranate peel, it can be observed that the scientific evidence has increased over the last two years.

A previous study indicated that the combination of green technologies (US, MW, high pressure, and supercritical carbon dioxide) with enzymatic pre-treatment could be a good tool to enhance polyphenols extraction from pomegranate peel. Recent research has indicated that a higher phenolic compounds yield was obtained from pomegranate peel using an enzymatic pre-treatment (Viscozyme^®^) followed by MAE than when conventional solvent extraction, EAE, or MAE was used [65]. Other authors have indicated that the optimum conditions of enzymatic pre-treatment and US technology was 41 min, 1.3% Viscozyme concentrationtion, and incubation for 1.8 h at 45 °C, obtaining extracts with a TPC of 20 mg GAE/g [48]. On the other hand, the pre-treatment enzymatic extraction did not improve the extraction yield when high pressure technology was applied to obtain punicalagin rich extracts [66]. With regard to enzyme-assisted supercritical fluid extraction process, it can be said that a high content of individual phenolic acids such as vanillic, ferulic, and syringic (108, 75 and 88 μg/g of extract, respectively) were found in the extracts. These phenolic acids were extracted thanks to enzymatic-assisted tecnology using pectinase, protease, cellulase, alcalase, and viscozyme [67]. Not only have promising results been obtained in the enzymatic extraction of pomegranate peel, but there is also evidence that it works as a pre-treatment in the extraction of bioactive compounds from other F&V byproducts [68]. More studies are needed to obtain the optimum conditions, depending on the raw material and the target compounds.

**Table 3:** Enzymatic assisted conditions (enzyme, pressure, time, temperature) for the extraction of bioactive compounds from pomegranate peel.

Byproduct Characteristics	Combined with	P (MPa)	Enzymes	Inactivation Enzymes	Solid/Liquid Ratio	t (min)	T (°C)	Extract Characterization	Ref.
Drier (40 °C) cv and particle size information NA	High pressure	300	4% (vol) of pectinase 4% (vol) of cellulase	Water bath 90 °C 5 min Later ice bath	1.6:100	15	NA	TPC Individual Ph	[66]
Dried Room Temperature Particle size < 0.178 mm cv information NA	Supercritical carbon dioxide	NA	Pectinase, protease, and cellulase (25:25:50)	Water bath 90 °C 5 min Later ice bath	2.8–3.9% of enzymes in the solvent	60–120	35–60	TPC	[67]
Drier (45 °C 48 h) cv and particle size information NA	Ultrasounds (Bath 40 kHz)	NA	Viscozyme^®^ concentration 0–2 mL/100 mL solvent	NA	1:10	0–60	30–50	TPC, TFC, AOX (DPPH)	[48]
Dried (more information NA) cv and particle size information NA	Microwave (300, 400 and 600 W)	NA	Viscozyme^®^ concentration 0.6% (*v*/*w*) pH 4.5 and 40 °C	NA	1:20, 1:30, 1:40 g/mL EtOH 30% acidified	90–150	NA	AOX (FRAP and CUPRAC), TPC	[65]

NA: Data not available; cv: cultivar; Ph: individual phenolics compounds; TPC: total polyphenolic content; AOX: total antioxidant capacity; TFC: total flavonoid content.

## 5. Pomegranate Peel Byproducts Incorporation Techniques

### 5.1. Powders/Flours

Pomegranate peel powder/flour is commonly acquired by drying and grinding until obtaining the desired particle size. Similar drying technology applied to edible fruit and plant material could be used in F&V byproducts to avoid undesirable bioactive compound changes [69]. The most common drying technologies are convective drying, sun-drying, MW drying, and freeze-drying in which key variables should be optimized (for instance, temperature and time). Moreover, spray-drying is commonly catalogued as a good tool for byproducts drying. This powder could be applied as a solid ingredient for the fortification of different products such as meat-based, F&V-based, and bakery products (Section 6) since this material presented high dietary fiber and techno-functional properties (high water- and oil-holding capacity, and low water absorption) in previous studies [70]. Similarly, powders can be obtained from liquid extracts after bioactive compounds extraction using different technologies such as freeze-drying or spray-drying [71]. Such technologies are included in the section on encapsulation due to the need for different processes to be carried out (Section 5.3).

### 5.2. Liquid Extracts

With pomegranate peel powders obtained as previously detailed, extraction techniques with different solvents can be used, including those reported in this review. These liquid extracts are not suitable for direct incorporation into the different food matrixes, except when the solvents may be classified as a food ingredient (e.g., water). Therefore, these solvents must be removed through evaporation. Once they have been evaporated, drying should be carried out (for instance convective or freeze-drying) to later redissolve it in water, as the most common liquid. In this way, the liquid extract is ready to be incorporated into the matrixes at different solid–liquid ratio, as observed in Section 6. In addition, liquid extracts can be used to obtain coatings, and can be encapsulated by different carriers and techniques, as outlined in Section 5.3.

### 5.3. Encapsulation

Encapsulation is a means to protect sensitive key bioactive compounds found in the food industry byproducts against undesirable heat, oxygen, light, and pH conditions [72]. The process needs a carrier agent and a technique to create the protective capsules. Different techniques may be used for the encapsulation of target compounds from F&V byproducts, such as spray-drying, freeze-drying, complex coacervation, and ion gelation [73], among others. Spray-drying is the liquid food drying method and has been widely used to obtain powders from F&V juices [69,74,75,76]. Currently, the transformation of F&V byproduct extracts (liquid) into powders using a spray-drier (the extracts are sprayed into a hot air chamber) has garnered attention because the process is complex, although this technique is one of the fastest, cheapest, and more reproducible, despite its complexity. In lyophilization as well as in spray-drying, a solution, dispersion, or emulsion is first obtained depending on the encapsulating agent and the active compound. The first step of freeze-drying-based encapsulation consists in creating an emulsion between the carriers and the target compounds, followed by a conversion into microcapsules by applying the freeze-drying technique [77], which consists of water removal by sublimation (primary drying) and secondary drying. Table 4 shows the main technologies (spray-drying, freeze-drying, double emulsion, and ion gelation) and the carriers used to encapsulate target bioactive compounds from pomegranate peel. It can be seen that there is an interest in using novel carriers such as citrus byproducts.

In addition, other technologies were applied for other pomegranate byproducts, such as complex coacervation, to obtain encapsulated pomegranate oil rich in punicic acid [96]. Complex coacervation is a liquid–liquid phase separation phenomenon that consists between oppositely charged biopolymers through electrostatic interaction, and this technique is increasingly used in the food industry due to its high encapsulation efficiency and optimal processing conditions [97]. After encapsulation processing, the encapsulated material presents the characteristics to be incorporated in other matrixes.

## 6. Potential Applications in the Food Industry

Pomegranate peel (in powders, liquid extract, and/or encapsulated, among others) have been reported in several food matrixes [98] such as F&V-based (Table 5), meat-based [15], fish-based [99,100], oil [101], dairy-based [102], confectionary [103], and baking products [82,104,105], among others. Packaging evidence have been reported by other authors, which has proven to be a good tool to preserve foods without altering their composition [106].

Since the bibliography on the incorporation of pomegranate byproducts into different food matrixes is extensive, this review has been focused on the scientific evidence related to the use of pomegranate peel byproducts during F&V handling and processing in the form of fresh whole, fresh-cut, minimally processed F&V, and beverages. Table 5 includes information about the characteristics of pomegranate peel byproducts (drying technique, particle size, and cultivar), extraction technique (US, maceration), incorporation method (liquid extracts, coating, dipping), and benefits tested after its incorporation (shelf life, bioactive compounds fortification). In the following sections, more specifications related to F&V based products are detailed.

### 6.1. Fresh Whole F&V

In this case, more than 15 types of evidence have been found, in which pomegranate peel extracts were incorporated in different F&V (Table 5), being >25% incorporated into citrus fruits. The incorporation of pomegranate peel extract as a postharvest technique in fresh whole F&V has been reported in ~90% of the included studies. A coating enriched with pomegranate peel extract is described in 42% of them, the control formulation in which the extracts were added being chitosan and alginate solutions. Additionally, scientific evidence related to preharvest application is reported (pomegranate peel atomization in tomato leaves and the incorporation of the soil in a sage herb field). Table 5 shows specific information related to the drying technique, particle size, and cultivar of pomegranate; the extraction technique; the extracts formulation and incorporation method (atomization, liquid extracts, coating, dipping); and the main results obtained by the authors.

### 6.2. Minimally Processed, or Fresh-Cut F&V

Since fresh-cut F&V usually present a short shelf life mainly due to enzymatic browning, dehydration, and microbial growth, it is necessary to look for innovative tools to preserve its quality and safety. Table 5 shows the scientific evidence in which pomegranate peel extracts were used in minimally processed or fresh-cut F&V. There is a need to focus on the different ways of incorporating extracts into other fresh-cut F&V, and salads (for instance, baby leaves and younger plants such as sprouts or microgreens). There is a lack of knowledge on the effect of pomegranate peel extracts on vegetable commodities.

### 6.3. F&V Based Beverages

The fortification of F&V based beverages with bioactive compounds has been recently reviewed and reported [8]. The goal of the fortification with target compounds could be to enhance functionality (high content of polyphenols and other compounds) and/or techno-functional properties (color maintenance, sensory quality, inhibition of microbial growth). Moreover, if the key biocompounds have been extracted by green technologies from F&V byproducts, their incorporation replaces or reduces synthetic additives. Table 5 shows the incorporation of pomegranate peel extracts in F&V juices as an alternative to enhance quality parameters. Future research should be focused on the fortification of other F&V-based matrixes such as cold/hot/dried soups and culinary sauces with pomegranate peel. For instance, a previous study indicated that the incorporation of horticultural byproducts improved the quality and shelf life of a kale pesto sauce [107].

**Table 5:** Application of pomegranate peel in fresh fruit and vegetable, minimally processed fruit and vegetable, and beverages.

	Matrix	Pomegranate Peel Byproduct	Extraction	Incorporation Method	Benefit	Ref.
**Fresh whole F&V (pre- and postharvest)**	Tomato	Drier (50–60 °C, 72 h) Fine powder (more information NA) cv information NA	Ratio 3:10 EtOH 48 h + evaporator (65 °C) + re-dissolved in sterile distilled water (0.05%, 0.5%, 1% and 5% *w*/*v*)	Preharvest. Tomato plants were sprayed in the leaves (bacteria inoculation) with the aqueous extract + 24 h drying	Antibacterial activity at least 15 days Replacing, reducing, or even alternating treatments involving copper compounds	[108]
	Sage herb	Air dried (more information NA) Grinder (more information NA) cv information NA	1:10 solid–liquid ratio in water or EtOH 80% 24 h + evaporator + water dilution	Preharvest. Added in the soil (2, 4, and 6 g per plot)	Higher dry mass and essential oils Inhibition of free radical scavenging	[109]
	Olive	Oven drier (40 °C) Powder home grinder (more information NA) Wonderful cv	120 g/L EtOH solvent (50 and 80%) + 1% Citric acid	Postharvest. Treatment of 1 × 1-mm injuries and inoculated (*C. acutatum*) by 10 µL of pomegranate peel extract (12, 1.2, or 0.12 g/L)	Reduction of fungal and bacterial population	[110]
	Potato tubers	Air drier (28 °C, 10–15 days) Fine powder (more information NA) Baladi cv	1:10 solid–liquid (MetOH) 48 h 28 °C + evaporator + oven 50 °C 48 h	Postharvest. Wound (3 × 3 mm φ and deep) + inoculation (*F. sambucinum*) (24 h) + dipping (1.25, 2.5, 5, 10, and 20 mg/mL water) + air dried (2 h at 28 °C).	Antifungal activity on the mycelial growth and spore germination	[111]
	Strawberry	Drying and particle size information NA Dente di caballo cv	US 40 °C 80% A 3 min (3 on, 8 off) Ratio 1:10 (H_2_O 25%, propanol 25%, ethanol 25% and methanol 25%) + evaporator + Freeze-drier + re-dissolved in water	Postharvest. Immersion (30 s in a 2 L solution of pomegranate peel extract) + air-drying (1 h)	Extension of shelf life Substitution of synthetic pesticides	[112]
	Sweet cherry	Oven drier (40 °C) Particle size NA Mollar de Elche cv	EtOH solvent (50 and 80%) + 1% Citric acid +evaporator + Water dilution	Postharvest. Dipping (2 min) in the pomegranate extract (12, 2.4 or 1.2 g/L) + air drying (2 h, 28 °C) + storage at 1 °C	Inhibition of all fungal spore germination	[113]
**Fresh whole F&V (pre- and postharvest)**	Sweet cherry	Oven drier (40 °C) Fine powder < 470 µm cv information NA	1:8 solid–liquid ratio (Water 28 °C 24 h)	Postharvest. Immersion in pomegranate peel extracts (3 min 20 °C) + room temperature drying	Pomegranate peel extracts and calcium sulphate coatings, alone or in combination, decreased weight loss, decay, respiration rate, and increased acidity, firmness, ascorbic acid, DPPH, TPC, and TAC	[114]
	Apple	Oven drier (40 °C) Particle size NA Mollar de Elche cv	EtOH solvent (50 and 80%) + 1% citric acid + evaporator + water dilution	Postharvest. Wounds treated with 10 μL of pomegranate peel extract (12, 1.2 or 0.12 g/L) + inoculation (10 μL *P. expansum*)	Inhibition of fungal spore germination and decay of artificial inoculations	[113]
	Mango	Freeze drying (−45 °C, 94 h) Particle size and cv information NA	MetOH 45 °C 30 min + Bath US + evaporator + water dilution	Postharvest. Chitosan (2%) in 0.5% citric acid solution + Pullulan (2%) in water (50:50 ratios). During stiring: 1% glycerol + 5% of pomegranate peel extract (0.02 g/mL). Dipping for 2 min	Increase of firmness, TPC and AOX. Prolonged the shelf life	[115]
	Apricot	Drier (60 °C, 48 h) Particle size < 0.251 mm cv information NA	80% EtOH 25 °C + evaporator	Postharvest. Chitosan coating solution (1% chitosan in glacial acetic 1% + 0.8% glycerol + Tween 80 + 0.50, 0.75, and 1% pomegranate peel extract)	Reduction of % decay and weight loss. Maintenance of DPPH radical scavenging activity, ascorbic acid content, titratable acidity and firmness.	[116]
	Figs	Air dried few days (more information NA) Pulverized (more information NA) cv information NA	Alcoholic buffer (EtOH 50%)	Postharvest. Alginic acid: agar (70:30) + 0.25 and 0.5% pomegranate peel extract Dipping in the coating solution + coating gelation	Prolonged the shelf life	[117]
	Dates	Drier (48 °C, 52 h) Ground peels (more information NA)cv information NA	EtOH 70% + evaporator + Water dilution	Postharvest. 1% Chitosan, 1% nanochitosan or 1% pomegranate peel extract in 1% glacial acetic	Growth inhibition of any fungal spore after 48 h of coating.	[118]
	Citrus	Hot air drier (50 °C, 48 h) Particle size 0.250 mm cv information NA	2.5:10 Solid–liquid ratio (Ac, EtOH, MetOH, H_2_O, DMSO) + shaking (6 h) + re-extracted with water evaporation	Postharvest. Immersion of wounded lemons (2 × 1 mm long and wide tip) in pomegranate peel extract (pre-infection and post-infection with *P. digitatum*) + air drying	Prevention and control of *P. digitatum*	[119]
**Fresh whole F&V (pre- and postharvest)**	Grapefruit	Oven drier (40 °C) Particle size information NA Mollar de Elche cv	EtOH solvent (50 and 80%) + 1% citric acid evaporator + water dilution	Postharvest. Wounds treated with 10 μL of pomegranate peel extract (12, 1.2 or 0.12 g/L) + inoculation 10 μL *P. digitatum* and *P. italicum*	Inhibition of all fungal spore germination and decay of artificial inoculations	[120]
	Lemon	Oven drier (40 °C) Particle size information NA Mollar de Elche cv	EtOH solvent (50 and 80%) + 1% citric acid + evaporator + water dilution	Postharvest. Wounds treated with 10 μL of pomegranate peel extract (12, 1.2 or 0.12 g/L) + inoculation 10 μL *P. digitatum* and *P. italicum*	Inhibition of all fungal spore germination and decay of artificial inoculations	[113,120]
	Mandarin	Drier (70 °C, 48 h) Ground peels (more information NA) Shirine Shahvar cv	0.25:10 solid–liquid ratio (60% EtOH + 0.1% citric acid)	Postharvest. Wounded (1 × 2 mm φ and depth) + dipping 1 min in pomegranate peel extract concentrations (25, 50, 75, 100%) + inoculation (*P. italicum* and *P. digitatum*) + drying	Reduction of % infected wound and lesion φ (75% or/and 100% extract). Increase of TPC, TFC, and PAL activity (75% or/and 100% extract)	[121]
	Orange	Drier (35 °C, 2 days) Particle size NA Gabsi cv	1:10, 0.6:10, 0.3:10 solid–liquid ratio (MetOH or Water) + evaporated + drying (40 °C or freeze-drying) + re-dissolved in water	Postharvest. Chitosan coating solution (1% chitosan in glacial acetic 1% + 0.5% Locust bean gum + 20% glycerol + 7, 18, and 36% dry waster/MetOH pomegranate peel extract). Wounded oranges (4 times: 3 × 3 mm φ × deep) + Inoculation (20 μL of a *P. digitatum)* + drying + dipping in different coating solutions (2 min)	Controlled growth of *Penicillium digitatum* Reduction of postharvest decay	[122]
	Orange	Oven drier (40 °C) Particle size NA Mollar de Elche cv	EtOH solvent (50 and 80%) + 1% citric acid + evaporator + water dilution	Postharvest. Wounded oranges (3 times 2 × 2 mm φ and deep) + 20 μL pomegranate peel extract (12 g/L) + Inoculation (20 μL of a *P. digitatum)* + 1% citric acid + drying	Enhanced defense pathways (antibiotic biosynthesis)	[123]
**Fresh whole F&V (pre- and postharvest)**	Guava	Drier (60 °C, 72 h) Particle size 0.420 mm Bhagwa cv	1:10 solid–liquid ratio (80% EtOH) + evaporation	Postharvest. Chitosan (1% chitosan in glacial acetic 1% + 0.75% glycerol) and alginate solution (2% alginate + 10% glycerol + 2% calcium chloride) with 1% pomegranate peel extract	Preserved quality for 20 d under refrigeration	[124]
	Capsicum	Drier (60 °C, 72 h) Particle size 0.420 mm Bhagwa cv	1:10 solid–liquid ratio (80% EtOH) + evaporation	Postharvest. Chitosan (1% chitosan in glacial acetic 1% + 0.75% glycerol) and alginate solution (2% alginate + 10% glycerol + 2% calcium chloride) with 1% pomegranate peel extract	Inhibition of microbial growth. Preserved sensory quality. Extension of shelf life up to 25 d at 10 °C	[125]
	Pear	Drier (60 °C, 72 h) Particle size 0.420 mm Bhagwa cv	1:10 solid–liquid ratio (80% EtOH) + evaporation	Postharvest. Chitosan (1% chitosan in glacial acetic 1% + 0.75% glycerol) and alginate solution (2% alginate + 10% glycerol + 2% calcium chloride) with 2% pomegranate peel extract	Lowered the cell wall degrading enzymes activity (firmness preservation)	[126]
**Fresh-cut/Minimally processed F&V**	Fruit salad: nectarine and pineapple in cubes covered with fructose syrup	Oven drier (38 °C, 48 h) Particle size 500 mm Wonderful cv	Powder	2.5–5% (*w*/*v*) of pomegranate peel powder at the container bottom	Inhibition of mesophilic bacteria, total psychrotrophic microorganisms, yeasts, and lactic acid bacteria No negative effect on sensory characteristics	[127]
	Fresh-cut pear, apple and melon (plugs)	Oven drier (40 °C) Particle size NA Mollar de Elche cv	EtOH solvent (50 and 80%) + 1% citric acid + evaporator + water dilution	Inoculated plugs were dipped (10 min, 150 rpm) + dried (25 °C 30 min)	Reduction of *Listeria monocytogenes*	[128]
	Fresh-cut Golden apple wedges: thickness 30-mm and 30 g	Drying and particle size information NA Dente di cavallo cv	Pulsed UAE (10 min, <50 °C, 1:40, 26 kHz, 200 W, 40% A, 50% duty cycle) + encapsulation with pectin from citrus peel by spray drying	Enrichment with microencapsulates reconstituted in water 1:1	Reduction of enzymatic browning. Color preservation	[129]
**Beverages**	Carrot juice	Oven drier (40 °C) Grounded in a colloid mill (more information NA) cv information NA	High pressure-assisted extraction	5 mg pomegranate peel extract per mL of carrot juice	Improvement of microbiological safety and AOX during storage. Color preservation	[130,131]
**Beverages**	Apple juice	Oven drier (55 °C, 12 h) Particle size and cv information NA	Maceration extraction (1:50, 80% EtOH 1 h shaking)	Different% of pomegranate peel extract (0–2%)	Enhancing sensory quality and AOX. Low toxicity with 1% of pomegranate peel extract	[132]
	Kiwi juice	Information NA	Commercial pomegranate extract (PureBulk, Roseburg)	Extract incorporation (180 μg/mL kiwi juice) + US bath (40 kHz, 180 W, 20 °C, 10–30 min)	US and pomegranate extract combined treatment: higher reductions on yeast and molds	[133]
	Red wine	Green decoction: Boiled in water 60 min (1:40) Freeze-drying of the extract Wonderful cv	Powder	Purification to obtain the tannins. 8 analyzed tannins (1 g L^−1^ wine solution)	Increase of protein stability Increase of color stability Reduction of sulfites	[134]
	Symbiotic drink powder	Hot oven (40 °C, 48 h) Particle size Kitchen-miller (more information NA) cv information NA	Ethanolic extract (80%; 1:15) + evaporator + Freeze-drier	Formulation: beetroot peel extract powder (3%), pomegranate peel extract powder (1%), grape pomace extract powder (1.5%), quince seed gum (0.5%), stevia (4%), mint (0.1%) and water (89.9%). Pasteurization: 72 °C, 90 s	Maintenance of *L. casei* viability of the recommended level of 10^−7^ CFU/g	[135]

NA: Data no available; A: amplitude; cv: cultivar; TPC: total polyphenolic content; TFC: total flavonoid content; AOX: total antioxidant capacity; TAC: total anthocyanin content; PAL: phenylalanine ammonia-lyase.

## 7. Conclusions and Future Perspectives

The research community and the food industry are quite interested in the use of (techno)-functional bioactive compounds from pomegranate byproducts in different food matrixes to reduce the use of synthetic additives and to develop ‘clean label’ products. However, the optimal extraction technique greatly depends on the raw material and conditions (cultivar, moisture, drying technology, particle size, etc.), so specific parameters should be recommended after a proper evaluation, on which more studies are needed. There is a lack of important information about the main characteristics of pomegranate peel, making it more difficult to have a more definitive conclusion on the optimal conditions of their bioactive compound extraction. Considering the three green extraction technologies included in this review, more than 80% of the evidence is focused on ultrasound-assisted technology. Therefore, more research on enzymatic and microwave-assisted methods, and their combinations, should be carried out. The combination of enzyme-assisted treatment with other green technologies usually increases the yield, shortening the extraction time. However, further research is still needed to optimize such combined treatments. In addition, a review of other green technologies for the extraction of bioactive compounds from pomegranate byproducts should be of interest to the research community, as well as other pomegranate byproducts such as seeds or arils. In future studies, the energy efficiency/consumption, the cost, and the environmental impact leading to a sustainable extraction of the key biocompounds must be evaluated. Additionally, predictive models are needed to optimize the phytochemical extraction and help in decision-making.

## Figures and Tables

**Table 2:** Microwave conditions (power parameters, solvent, time, temperature) for the extraction of bioactive compounds from pomegranate peel.

Byproduct Characteristics	Power (W)	Pressure	Solvent	Solid/Liquid Ratio	T (min)	T (°C)	Other Information	Extract Characterization	Ref.
Milled frozen (more information NA) Wonderful cv	2000, 4000, 6000	Vacuum 355 mbar	NA	1:10; 1:25; 1:40	10, 50, 90	40, 50, 60	Industrial-type MAC-75 multimode MW extractor	TPC, AOX (DPPH)	[57]
Drier (40 °C, 48 h) Particle size ~0.1 mm cv information NA	100, 173, 350, 527, 600	75 bar	H_2_O EtOH (50–70%) MetOH (50–70%)	1:10; 1:17.3; 1:35; 1:52.7; 1:60	0.5–15	NA	Multiwave closed MW system	TPC, AOX (DPPH), Pn, EA	[58]
Air-dried (4–5 days) Particle size 0.75–2 mm cv information NA	470–800	NA	EtOH (50%)	1:10	20	41–72	Home-made setup consisting of MW oven	TPC, GA, EA, Pn, HMF	[59]

NA: Data not available; Pn: punicalagin; TPC: total polyphenolic content; AOX: total antioxidant capacity; EA: ellagic acid; GA: gallic acid; HMF: hydroxymethylfurfural.

**Table 4:** Main technologies used to encapsulate target compounds from pomegranate peel.

Technology	Carriers	Target Compound/Activity	Ref.
Spray-drying	Maltrodextrin	F-TPC, UPLC-TPC, Pn, EA, P, GA	[78,79]
	Maltrodextrin + others: Tween 80 (99:1); Skimmed milk powder (50:50); Whey protein isolate (50:50); Gum arabic (50:50)	NA (Yield/Stability)	[28,80]
	Skimmed milk power	NA (Yield/Stability)	[28,80]
	Orange juice byproduct	F-TPC, DPPH	[81,82]
	Maltodextrin/Pectin	TPC, Pn, EA	[83]
	Whey protein	Pn, EA, P, GA	[79]
	Arabic gum	Pn, EA	[84]
	Chitosan	Pn, EA	[84,85]
	Pectin	Pn, EA	[84]
	Modified starch	Pn, TPC, HTC, DPPH	[86]
	Alginate	NA (Yield/Stability)	[85]
Freeze-drying	Soy phosphatidylcholine liposomes	Pn, EA, rutin, epifallocatechin, TPC	[87]
	Maltodextrin (5 and 10%) and b-cyclodextrin (5 and 10%).	F-TPC, FRAP	[38]
	*Prunus armeniaca* gum exudates	FRAP, DPPH	[88]
	Chitosan	FRAP, DPPH	[88]
	Maltrodextrin	TPC, TFC, Pn, EA, FRAP, DPPH	[89]
	Maltodextrin and calcium alginate	ANCs, FRAP, DPPH	[90]
	Maltodextrin and soy lecitin	NA (Yield/Stability)	[91]
Double emulsion	Water^1^ in Oil in Water^2^: Water^1^ (ethanolic solutions) in Oil (castor, soybean, sunflower, medium chain triglyceride and orange) in Water^2^ (aqueous solution with Tween^80^)	NA (Yield/Stability)	[92]
Ion gelation	Chitosan gel (1%):gelatin 2:1	F-TPC, DPPH	[93]
	Spirulina	TPC, DPPH	[72]
	Microalgae	EA	[94]
	Chitosan + others: Dialdehyde guar gum Gelatin-based materials	F-TPC, DPPH	[95]

NA: Data not available; cv: cultivar; EA: ellagic acid; F-TPC: total polyphenolic content by Folin assay; UPLC-TPC: total polyphenolic content by UPCL; TFC: total flavonoid content; Pn: punicalagin; P: punicalin; GA: gallic acid; HTC: hydrolysable tanin content; ANCs: anthocyanins.

## Data Availability

No new data were created or analyzed in this study. Data sharing is not applicable to this article.
